# Integrative Analysis of Metabolome and Transcriptome Reveals the Role of Strigolactones in Wounding-Induced Rice Metabolic Re-Programming

**DOI:** 10.3390/metabo12090789

**Published:** 2022-08-25

**Authors:** Ling Liu, Kang Li, Xiujuan Zhou, Chuanying Fang

**Affiliations:** 1 Sanya Nanfan Research Institute of Hainan University Hainan, Yazhou Bay Seed Laboratory, Sanya 572025, China; 2 College of Tropical Crops, Hainan University, Haikou 570288, China

**Keywords:** strigolactones, wounding, jasmonates, metabolic response

## Abstract

Plants have evolved mechanisms to adapt to wounding, a threat occurring separately or concomitantly with other stresses. During the last decades, many efforts have been made to elucidate the wounding signaling transduction. However, we know little about the metabolic re-programming under wounding, let alone whether and how strigolactones (SLs) participate in this progress. Here, we reported a metabolomic and transcriptomic analysis of SLs synthetic and signal mutants in rice before and after wounding. A series of metabolites differentially responded to wounding in the SLs mutants and wild-type rice, among which flavones were enriched. Besides, the SLs mutants accumulated more jasmonic acid (JA) and jasmonyl isoleucine (JA-lle) than the wild-type rice after wounding, suggesting an interplay of SLs and JAs during responding to wounding. Further transcriptome data showed that cell wall, ethylene, and flavones pathways might be affected by wounding and SLs. In addition, we identified candidate genes regulated by SLs and responding to wounding. In conclusion, our work provides new insights into wounding-induced metabolic re-programming and the SLs’ function.

## 1. Introduction

Wound stress commonly occurs when plants suffer from various biotic attacks and abiotic stresses (e.g., wind damage). In a few minutes to several hours post wounding, a series of complex and fine-tuned responses occur, including reactive oxygen species (ROS) status [1], calcium (Ca^2+^) contents [2], gene expression levels [3], and metabolism [4,5,6]. Wounding-regulated natural compounds have been reported [7]. Plants’ metabolic responses to wounding are a complex process, including the change of a series of volatile and non-volatile metabolites. For instance, in various tropical agricultural species, wounding triggers the release of volatile organic compounds (e.g., methanol, hexenal, and acetaldehyde), which are produced at relatively low levels under normal conditions. Besides, the lipoxygenase pathway and lightweight oxygenated compounds are activated by wounding in 1~2 min [6]. A series of non-volatile metabolites also respond to wounding. For example, glucose and asparagine pile up in the wounded Mediterranean sclerophyllous tree, while the contents of valine and leucine decline. In addition, wounding plants produce a significantly higher content of secondary metabolites. Some of them are related to defense against biotic and abiotic stress and wound recovery, including quinic acid, quercitol, choline, N-acetyl group, and malic acid [8]. Herbivore attacks trigger the expression of flavonoid-related genes and the accumulation of flavonoids in tea leaves (*Camellia sinensis*), which further participate in the defense response against the tea green leafhopper [9]. In rice, herbivory and mechanical stimuli perturbed phenylamide and increased the accumulation of caffeoylagmatine [10]. However, the metabolic responses remain exploited through the metabolome lens.

Jasmonates (JAs), a class of oxylipins-derived phytohormone, respond to wounding dramatically. Wounding triggers notable increases (~25-fold) in jasmonic acid (JA) and jasmonyl isoleucine (JA-Ile) contents within 5 min [11]. The biosynthesis of JAs starts with the release of linolenic acid from phospholipids by phospholipases (PLs) [12,13,14]. Linolenic acid is further subject to oxidation reaction catalyzed by 13-lipoxygenases [15] and produces JA by a series of reactions. GH3 (GRETCHEN HAGEN)-family proteins (OsGH3.3/OsJAR2, OsGH3.5/OsJAR1) conjugated JA to Ile or other amino acids [16,17]. JAs biosynthesis and signaling transduction transcriptionally and metabolically signaling respond to wounding [18,19].

Plant adaptation is regulated by complicated phytohormone interactions [20,21]. In recent decades, the JAs pathway has been reported vital for wounding responses [11,22]. In addition, ethylene represses the expression of JAs synthesis-related genes, including a PLA1-type phospholipase gene *GY1*, to reduce JAs levels and promote mesocotyl and coleoptile growth [23]. JAs and ethylene interplay to regulate the biosynthesis of anthocyanins and flavones. Ethylene inhibits anthocyanins biosynthesis in red Chinese pear fruits, whereas JA induces anthocyanins and flavones biosynthesis, including velutin, luteolin, chrysoeriol, and apigenin. The branches of the JA-induced anthocyanins pathway are determined by ethylene [24]. Moreover, ethylene induces the accumulation of luteolin in *Matricaria chamomilla* [25]. However, we know little about how JAs interplay with other hormones to determine the metabolic responses to wounding.

Strigolactones (SLs) are a class of carotenoid-derived phytohormone containing more than 30 molecules [26]. Firstly, DWARF27 (D27), an all-*trans*/9-*cis*-β-carotene isomerase, catalyzes the reversible isomerization of all-*trans*-β-carotene into 9-*cis*-β-carotene, which cleaves at the C9′-C10′ double bond and produces 9-*cis*-β-apo-10′-carotenal and β-ionone under the catalyzation of a carotenoid cleavage dioxygenase CCD7 (also known as DWARF17, D17). Then, CCD8 (i.e., DWARF10, D10) converts 9-*cis*-β-apo-10′-carotenal into carlactone (CL), the precursor of canonical and non-canonical SLs [27]. DWARF14 (D14) interacts with DWARF3 (D3) and DWARF53 (D53) in the presence of SLs, leading to the ubiquitination and degradation of the nuclear-localized repressor D53 [28,29]. In addition to regulating plant architecture and adaptation to environmental stresses [30,31], SLs are vital for plant metabolism [32]. Recent studies suggest putative crosstalk between SLs and JAs [33]. SL-RNAi tobacco plants accumulate more SLs signaling repressors (SMXL6/7) and JAs. Further work revealed that SML6/7 directly interacts with and trigger the degradation of jasmonate zim-domain (JAZs), which are repressors of JAs signaling. Then, the released basic helix-loop-helix-leucine zipper transcription factor, MYC2, activates the JA signaling pathway and induces the contents of anthocyanins and phenolamides [33]. However, whether and how SLs participate in wounding signal transduction remains unclear.

Herein, we reported the metabolomic responses of rice under wounding stress. Using rice mutants of SLs biosynthesis (*d10*) and signaling (*d3*, *d14*), we characterized the role of SLs in metabolic re-programming under wounding. The contents of JA and JA-lle significantly increased in the SLs mutants after wounding, implying crosstalk between JAs and SLs. Transcriptome data and pathway analysis revealed that SLs might regulate flavones and ethyne pathways. Furthermore, we identified candidate genes through which SLs regulate metabolic responses to wounding.

## 2. Materials and Methods

### 2.1. Plant Materials

The SLs mutants were constructed in our previous work [34]. The rice plants were cultivated in Hainan University (Haikou, China, N 20°02′, E 110°11′). All the seeds were germinated for three days at 37 °C on filter paper soaked in distilled water and then planted in seedbeds. Subsequently, two-week-old seedlings were planted by hydroponic culture using Yoshida nutrient solution [35].

In metabolic and transcriptomic analyses, one-month seedlings were used and the leaves were sampled and extracted under the normal condition and after half an hour of mechanical wound. The rice leaves were cut for 5 cm along the main vein with scissors. One half of the cut leaf was sampled immediately as control, and the other was harvested 0.5 h after wounding as treated samples. The upper second and third leaves of three independent plants were harvested and combined into a biological replicate. Two biological replicates were collected for each genotype.

### 2.2. Metabolic Sample Preparation and Metabolite Profiling

Metabolic profiling was performed as previously described [32]. The freeze-dried leaves were ground into powder by using a grinder (MM 400, Retsch, Haan, Nordrhein-Westfalen, Germany) at 30 Hz for 1.5 min. Then, ~100 mg of powder was weighed, and 70% methanol-aqueous solution was added at 0.1 mg/mL. These samples were then extracted by ultrasonication at 40 Hz for 10 min. After centrifugation and filtration (SCAA-104, 0.22 mm pore size; ANPEL, Shanghai, China), the samples were quantified by the MRM method of LC-MS 8060 (Shimadzu, Kyoto, Japan) [36,37,38], setting the detection window to 120 s and the target scan time to 1.5 s. A total of 615 transitions were monitored. The Multiquant 3.0.2 was used to process the original data. We normalized the metabolites contents through divided the relative signal strengths of the metabolites by the strength of the internal standard (0.1 mg/L lidocaine) and then log_2_ transformed the values to further improve the normalization.

### 2.3. The Analysis of Differntially Accumulated Metabolites (DAMs)

Principal component analysis (PCA) was performed to compare the contents of the expressed metabolites profiles among the SLs mutants and wild-type rice before wounding and after wounding using the *PCAtools* package in R. Heatmaps of metabolites were generated using the *complexheatmap* package in R. The identification criteria of differential metabolites were |log_2_ (fold change)| > 1 and *p*-value < 0.05, which was calculated by univariate analysis (*t*-test). The volcano maps of SLs mutants and wild-type rice were used *ggplot*2 package in R and the interest metabolites were filtered according to the log_2_ (FC) and -log10 (*p*-value) of metabolites. The Venn plots of DAMs shared by SLs mutants and wild-type rice were obtained by using online website (http://www.bioinformatics.com.cn/static/others/jvenn/index.html, accessed on 20 March 2022). Then, the metabolites only regulated in SLs-mutants and wild-type rice were manually checked to filter the non-conforming metabolites.

### 2.4. RNA Sequencing

According to the protocol, we extracted the total RNA from the leaves with a TRIzol reagent (Cat# DP424, TIANGEN Biotech Co. Ltd., Beijing, China), and a 2100 Bioanalyzer (Agilent Technologies, Santa Clara, california, USA) was used to confirmed the integrity of the total RNA. Then, high-quality RNA samples (OD260/280 = 1.8 to 2.2, approximately, OD260/230 ≥ 2.0, RIN ≥ 8, > 1 μg) were used to construct the sequencing library. After purified polyA mRNA from total RNA using oligo-dT-attached magnetic beads, which were subjected to a fragmentation buffer. The first strand cDNA was synthesized using reverse transcriptase, random primers, and the short fragments, followed by second strand cDNA synthesis. Then, the synthesized cDNA was subjected to end repair, phosphorylation, and “A” base addition. Both sides of the cDNA fragments were added to the sequencing adapters. After PCR amplification of the cDNA fragments, the 150 to 250 bp target fragments were cleaned up. Paired-end sequencing on an Illumina HiSeq × Ten platform (Illumina Inc., San Diego, CA, USA) was performed.

### 2.5. RNA-Sequencing Data Analyses

Our pipeline consists of the following steps to analysis data: first, the raw data were processed by fastp v0.23.2 [39] with default settings to remove low-quality bases and sequencing adapters. Then, Hisat2 v2.1.0 [40] with default parameters was used to map the clean paired reads to the rice reference genome (MSU7.0). The mapped fragments for each gene were counted by featureCounts [41], and transcripts per million (TPM) were calculated. Genes with averaged TPM > 1 (samples = 16) were considered expressed. DEG analysis was performed with count tables in R v4.1.0 using DEseq2, and genes with a *q*-value < 0.05 and abs(log_2_fold-change) > 1 were classified as DEGs. The GO analysis was conducted with DEGs by using online David website (https://david.ncifcrf.gov/tools.jsp, accessed on 29 April 2022). The online KOBAS website (http://kobas.cbi.pku.edu.cn/kobas3/genelist/, accessed on 29 April 2022) was used for KEGG analysis. Then, R software was used to draw the graph.

### 2.6. Gene Network Analysis

Protein–protein interaction (PPI) network was identified using STRING database (https://string-db.org/, accessed on 30 April 2022) with *oryza sativa* as the reference to retrieve protein–protein interactions. The network file was visualized using Cytoscape (v3.7.2) [42] software to present a core and hub protein biological interaction.

### 2.7. MapMan Analysis

Differences in the expression of genes in different groups involved in each functional module were shown with MapMan (version 3.6.0 RC1, Berlin, Germany). All genes that could be annotated in regulatory pathways were tagged and their relative expression heatmaps were used to show the response of certain metabolic pathways to the wounding and SLs. TBtools (v1.098761) was used for heatmap of DEGs in each module [43].

### 2.8. Correlation Analysis of DEGs

We used the R software to calculate the correlation of DEGs and DAMs based on Pearson correlation coefficient. The correlation heatmap was drawn using R-package *pheatmap*. Correlation filtering is based on *p*-value, |*p*| > 0.8, which is considered highly correlated.

## 3. Results

### 3.1. HPLC-MS/MS-Based Quantitative Metabolomic Analysis

**Figure 1:**
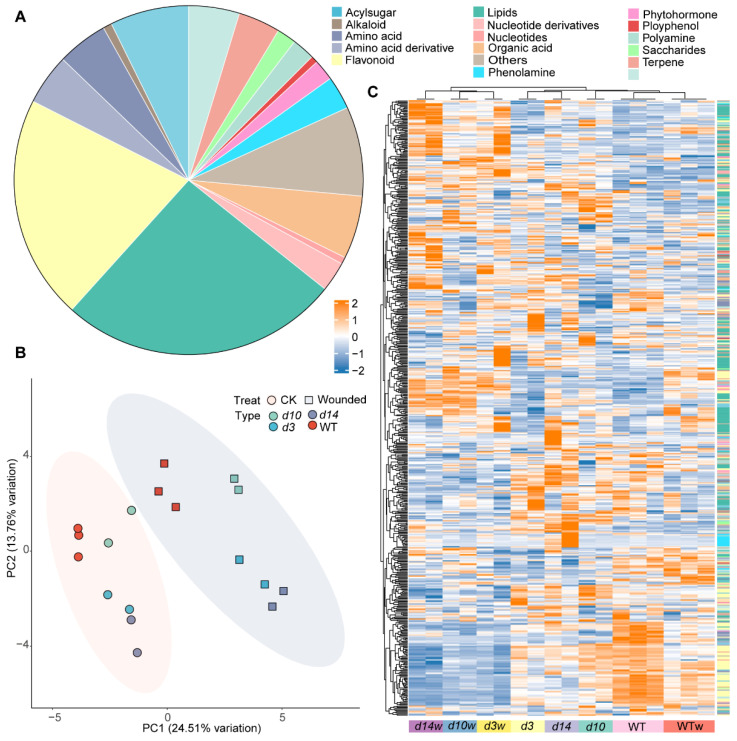
Metabolomic analysis of the SLs mutants and wild-type (WT) plants before and after wounding. (**A**) A total of 615 metabolites were detected in this study. (**B**) Principal component analysis (PCA) and (**C**) heatmap of the 615 metabolites in SLs mutants (*d10*, *d3*, and *d14*) and wild-type plants. PC1 and PC2 refer to the first and second principal components, respectively. WT means wild type; *d3*, *d10*, and *d14* represent mutants of *DWARF3*, *DWARF10*, and *DWARF14*, respectively. WTw, *d3w*, *d10w*, and *d14w* samples were collected 0.5 h after wounding.

To understand how metabolites respond to wounding and whether SLs participate in this progress, we performed a high-performance liquid chromatography–electro spray ionization–tandem mass spectrometry (HPLC–ESI–MS/MS) based widely-targeted metabolomic analysis using the mutants of SLs biosynthesis (*d*10) and signaling (*d*3 and *d*14). A total of 615 metabolites were detected, including both primary and secondary metabolites. Most primary metabolites belong to lipids metabolism (160), amino acids and derivatives (58), nucleotides and derivatives (21), organic acids (36), and vitamins (29). The detected secondary metabolites included 128 flavonoids, 24 terpenoids, and 45 acyl-sugars (Figure 1A). We performed a principal component analysis (PCA) to visualize the metabolites’ distribution in all samples. Samples from control and wounding conditions were clustered into two groups (Figure 1B), suggesting a remarkable metabolic re-programming upon wounding. Moreover, in each cluster, samples of the SLs mutants were distinguished from wild-type rice, implying that SLs affect metabolic signatures in rice (Figure 1B). Consistent with the PCA results, the heatmap clustering separated the wild-type and SLs mutants and wounded and unwounded. The metabolites content was clustered into two categories. The upper category showed no obvious pattern in all samples, while the lower category showed lower content in SLs mutants. In particular, some flavonoids decreased significantly after wounding in the SLs mutants. These results suggested that flavonoids may be involved in the SLs-mediated response to wounding (Figure 1C).

**Figure 2:**
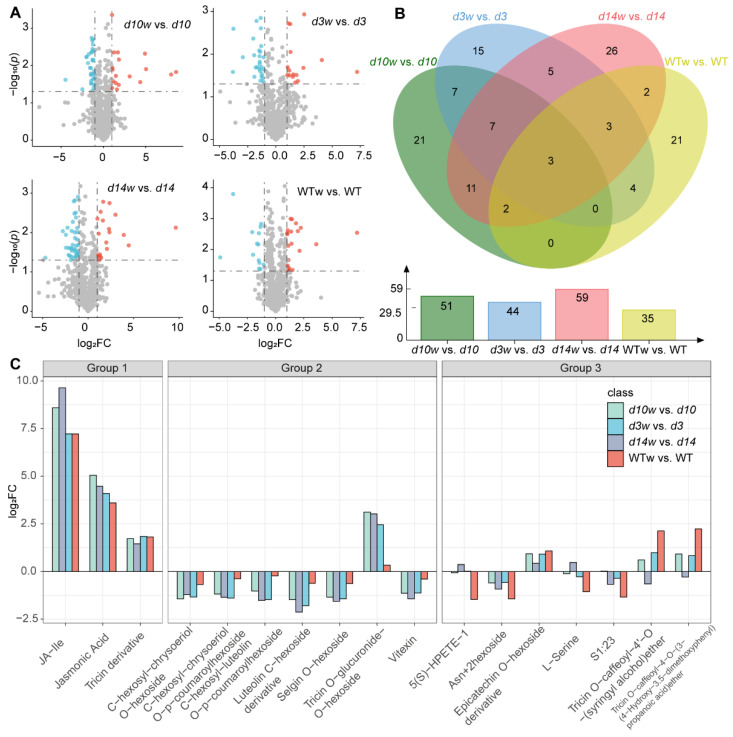
Schematic representation of differential accumulation metabolites (DAMs) between mutants and wild type. (**A**) DAMs in the SLs mutants (*d3*, *d10*, and *d14*) and wild-type plants. (**B**) Venn diagrams showing DAMs shared between the SLs mutants and wild-type plants. (**C**) The DAMs that accumulated in each group. WTw, *d3w*, *d10w*, and *d14w* samples were collected 0.5 h after wounding.

Next, we identified differentially accumulated metabolites (DAMs, |log_2_fold change (FC)| ≥ 1 and *p*-value < 0.05) upon wounding. In total, 51, 44, 59, and 35 wounding-responded DAMs were found in *d10*, *d3*, *d14,* and wild-type mutants, respectively (Figure 2A,B). In wild-type plants, JA-lle and JAs displayed the strongest responses to wounding, with a more than 147 and 11-fold increase, respectively. In addition, a series of compounds responded to wounding. In total, 8 out of 21 wounding-upregulated DAMs in wild-type mutants were flavonoids, including 5 tricin derivatives. Meanwhile, wounding depressed the production of 14 metabolites in the wild-type plants, including 4 lipids, 3 amino acids and derivatives, and 2 flavonoids. Nicotinamide-N-oxide and trigonelline were the most significant DAMs, with a more than 93% loss in content after wounding (Table S1).

In the SLs mutants, the content of four and 6six compounds increased and decreased upon wounding, respectively. Consistent with that in wild-type plants, JA-lle and JA boosted after wounding in the SLs mutants, with a more than 148- and 16-fold increase, respectively. Besides, the accumulation of two tricin derivatives increased after wounding. In addition, six flavones were downregulated by wounding with 51~77% loss in SLs mutants (Table S1). 

Then, we compared wounding-responded DAMs in the SLs mutants and wild-type plants. JA, JA-lle, and a tricin derivative (hereafter referred to as Group 1) showed similar patterns in each genotype (Figure 2C). On the other hand, we found that seen flavones are SLs mutants-only DAMs (Group 2); including vitexin, luteolin C-hexoside derivative, selgin O-hexoside, C-hexosyl-chrysoeriol O-hexoside, tricin O-glucuronide-O-hexoside, C-hexosyl-luteolin O-p-coumaroylhexoside, and C-hexosyl-chrysoeriol O-p-coumaroylhexoside. Moreover, 21 DAMs were only identified in wild-type plants (Group 3). These results implied that SLs function in rice metabolic responses to wounding

### 3.2. Transcriptome Analysis

To investigate the molecular basis of metabolic responses to wounding, we conducted an RNA-Seq study using leaves of each genotype before and after wounding. On average, we obtained 6.88 Gb clean reads for each sample, 97% of which could be mapped to the reference genome of rice (Table S2). In total, 19,791 genes expressed (averaged TPM  ≥  1) in 16 samples.

**Figure 3:**
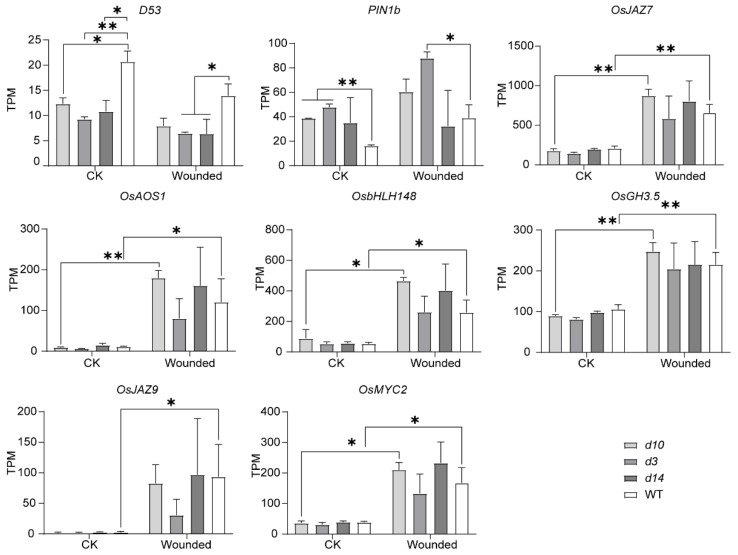
The transcripts per million (TPM) of SLs and JAs pathway-related genes in SLs mutants and wild-type plants before and after wounding. The data were represented as mean ± SD of two biological replicates. The Student’s *t*-test analysis indicated a significant difference (* *p* < 0.05, ** *p* < 0.01).

To test the reliability of the transcriptome data, we checked the expression of genes regulated by SLs or JAs (Figure 3). As reported by Jiang et al. [28], *D53* expressed at lower levels in the SLs mutants than in wild-type plants. In addition, the wounding induced JAs biosynthetic and JAs signaling genes, such as *OsJAZ7*, *OsJAZ9*, *OsAOS1*, *OsbHLH148*, *OsGH3.5*, and *OsMYC2*.

**Figure 4:**
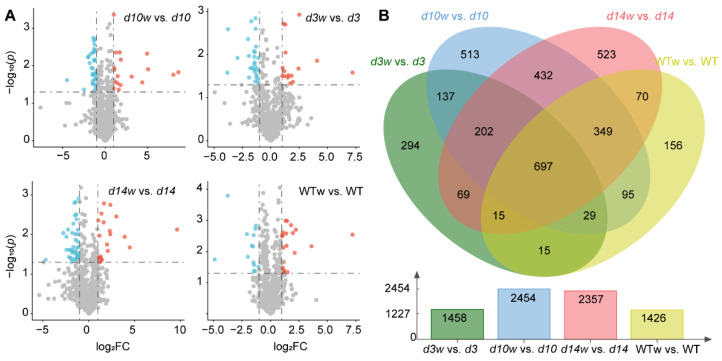
Schematic representation of differentially expressed genes (DEGs) between the mutants and wild type. (**A**) DEGs in the SLs mutants (*d3*, *d10*, and *d14*) and wild-type plants. (**B**) Venn diagrams showing DEGs shared by the SLs mutants and wild-type plants. WTw, *d3w*, *d10w*, and *d14w* samples were collected 0.5 h after wounding.

To identify genes responding to the wounding, we characterized differentially expressed genes (DEGs) based on |log_2_FC| ≥ 1 and *q*-value < 0.05. In wild-type plants, we identified 1242 wounding-upregulated and 184 wounding-downregulated DEGs (Table S3). A total of 1458, 2454, and 2357 DEGs were identified in *d3*, *d10*, and *d14*, respectively (Figure 4). Among them, the three mutants harbored 899 common DEGs, including 858 upregulated and 41 downregulated DEGs.

**Figure 5:**
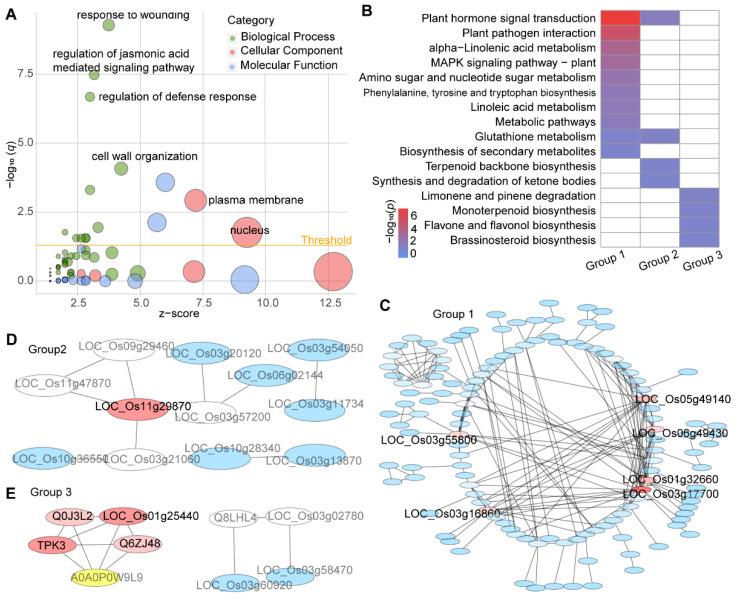
Enrichment analysis of DEGs in each group. (**A**) Functional annotation based on DEGs in Group 1. Results are summarized under three major functional classes: biological processes, molecular functions, and cellular components. (**B**) Significantly enriched KEGG pathways among DEGs in each group. The red and blue represent the *p*-values. Protein–protein interaction (PPI) network constructed by DEGs in each group (**C**–**E**).

Then, in order to find DEGs that were affected by the SLs pathway in responding to wounding, we compared wounding-responded DEGs in SLs mutants and wild-type plants. The SLs mutants and wild-type plants shared 697 DEGs (Group 1), significantly enriched in the gene ontology (GO) terms “response to wounding”, “regulation of jasmonic acid-mediated signaling pathway”, “regulation of defense response”, “cell wall organization”, “response to water deprivation”, “jasmonic acid biosynthetic process”, “intracellular signal transduction”, “oxylipin biosynthetic process”, “response to cold”, “response to salt stress”, and “response to abscisic acid” (Figure 5A, Table S4). Meanwhile, we identified 202 and 156 DEGs only in SLs mutants (Group 2) and wild-type plants (Group 3), respectively (Figure 4A). Group 2 was significantly enriched in “transcription factor activity, sequence-specific DNA binding”, “serine-type endopeptidase activity”, and “cellular response to heat”. Genes from Group 3 are likely to function in “growth factor activity” and “nucleosome” (Table S4).

### 3.3. Pathway Enrichment Analysis

We performed the Kyoto Encyclopedia of Genes and Genomes (KEGG) analysis with the DEGs (Table S5). The Group 1 DEGs were mainly from “plant hormone signal transduction”; “plant–pathogen interaction“; “alpha-linolenic acid metabolism”; “MAPK signaling pathway—plant”; “amino sugar and nucleotide sugar metabolism”, “phenylalanine, tyrosine, and tryptophan biosynthesis”; “linoleic acid metabolism”; “glutathione metabolites”; and “biosynthesis of secondary metabolites” (Figure 5B, Table S5). Group 2 mainly consisted of genes from “glutathione metabolism”, “terpenoid backbone biosynthesis”, and “synthesis and degradation of ketone bodies”. Group 3 genes were enriched in “limonene and pinene degradation”, “monoterpenoid biosynthesis”, “flavone and flavanol biosynthesis”, and “brassinosteroid biosynthesis” (Figure 5B, Table S5). 

Besides, we predicted the protein–protein interaction (PPI) network among DEGs using the STRING database [44]. In the PPI network constructed using Group 1 genes, *LOC_Os03g17700* (*OsMPK3*), *LOC_Os05g49140* (*OsMPK7*), *LOC_Os06g49430* (*OsMPK12*), *LOC_Os01g32660* (*OsMKK6*), *LOC_Os03g55800* (*OsAOS1*), and *LOC_Os03g16860* (*OsHSP71.1*) were the hub genes involved in response to wounding in each genotype (Figure 5C). *OsMPK3, OsMPK7, OsMPK12,* and *OsMKK6* have been reported to be associated with biotic and abiotic stress in rice [45]. The *OsAOS1* protein catalyzes the formation of JA [46]. In addition, *LOC_Os11g29870* (*OsWRKY72*), which depresses JA production by transcriptionally repressing *OsAOS1*, apparently interacts with Group 2 DEGs (SLs mutants-only group) [47]. Meanwhile, *LOC_Os01g25440* and *LOC_Os03g02780* were potential interactors of Group 3 DEGs (wild-type-only group) (Figure 5D,E).

### 3.4. Differentially Expressed Metabolism-Related Genes

We mapped DEGs to the software MapMan’s MSUv7.0 database for gene annotation and functional classification.

**Figure 6:**
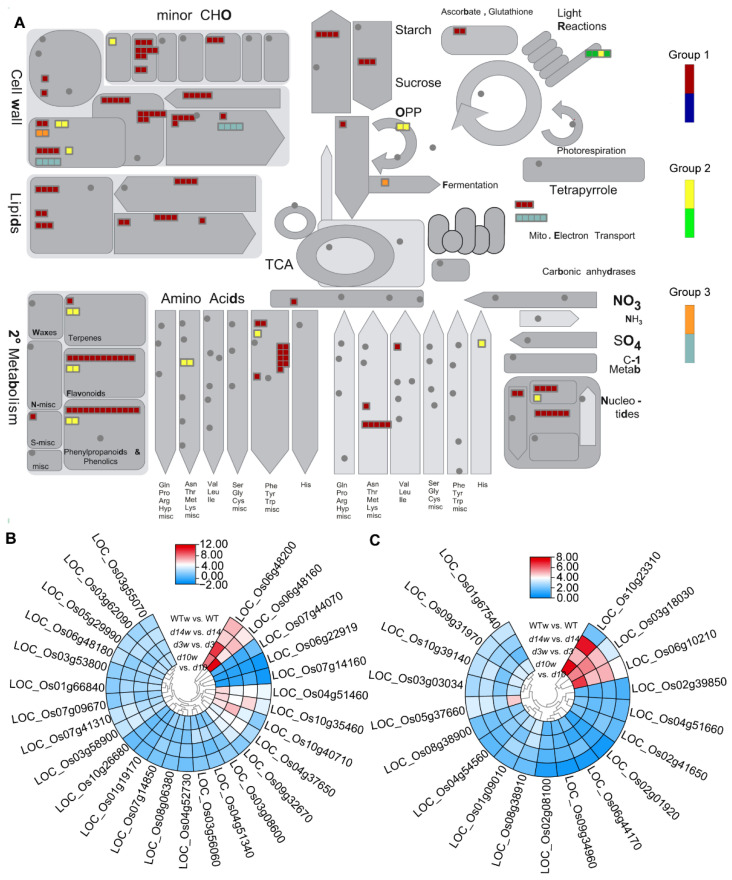
MapMan analysis based “metabolic overview map” of DEGs in three groups. (**A**) Schematic diagram of metabolic overview categories. The small heatmap in each section of the diagram shows the DEGs mapped to the pathway and a small square indicates a transcript. Red, orange, and yellow indicate upregulated genes in each group; blue, light sea green, and green mean downregulated genes in each group. (**B**,**C**) DEGs related to (**B**) cell wall and (**C**) secondary metabolism. Heatmaps of DEGs were drawn using the log_2_ (FC) value obtained from the pairwise comparison of samples. Red and blue indicate upregulation and downregulation, respectively.

To identify candidate genes of wounding-regulated metabolic pathways in the three groups, we obtained “metabolic overview” profiles of DEGs using MapMan (Figure 6A). The heatmap distribution showed that wounding-responded DEGs were mainly enriched in cell wall and secondary metabolism. Lots of cell wall genes were significantly upregulated in each genotype. Similarly, terpenes, flavonoids, and phenylpropanoids and phenolics genes were upregulated in Group 1 and Group 2.

Then, we performed a heatmap analysis of DEGs in the functional modules of cell wall and secondary metabolism. We identified 27 and 18 DEGs associated with cell wall and secondary metabolism, respectively (Figure 6). Among them, 24 and 15 had similar fold changes in the SLs mutants and wild-type plants. Meanwhile, the other six genes were prone to larger increases following the wounding in the SLs mutants than in wild-type plants (Figure 6B,C), implying the involvement in the SLs-mediated responses to wounding.

**Figure 7:**
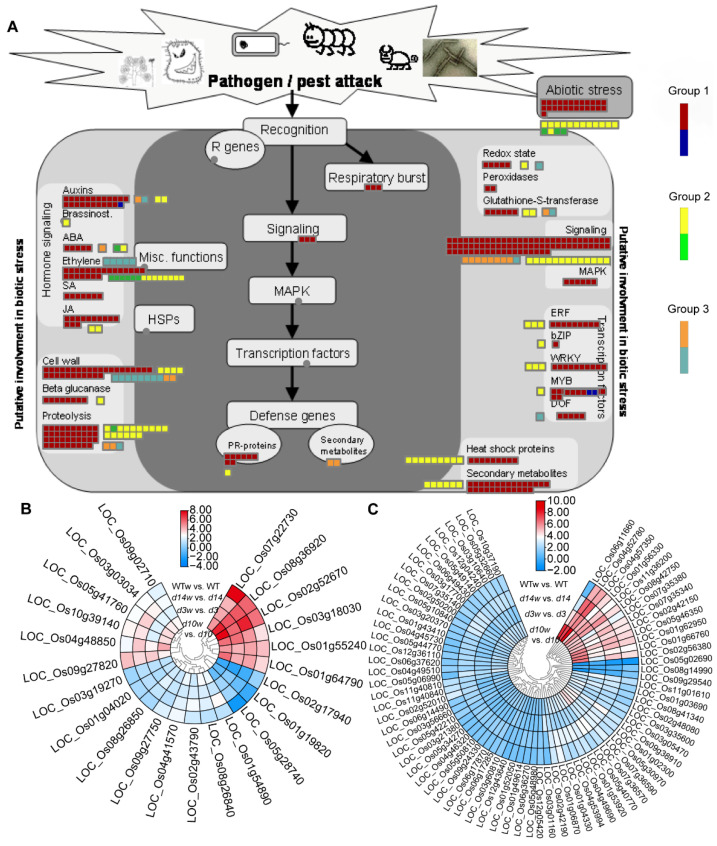
MapMan analysis based “biotic stress pathways” of DEGs in three groups. (**A**) The schematic diagram of biotic stress categories. The small heatmap in each section of the diagram shows the DEGs mapped to that pathway, and a small square indicates a transcript. Red, orange, and yellow indicate upregulated genes in each group; blue, light sea green, and green means downregulated genes in each group. (**B**,**C**) DEGs related to ethylene metabolism (**B**) and signaling pathway (**C**). Heatmaps of DEGs were drawn using the log_2_ (FC) value obtained from the pairwise comparison of samples. Red and blue indicate upregulation and downregulation, respectively.

Then, we analyzed DEGs of biotic stress pathways in all three groups with the overview map of “biotic stress” (Figure 7). Most DEGs were enriched in the ethylene, cell wall, proteolysis, redox state, glutathione-S-transferase, and signaling. Although most genes expressed at higher levels after wounding, ethylene and cell wall genes in Group 3 were downregulated by wounding (Figure 7A). As ethylene and signaling pathways occupied the most genes, we check and found 23 and 73 DEGs related to ethylene and signaling pathways, respectively. Although 21 ethylene-related DEGs shared similar patterns between wild-type plants and the mutants, the responses of LOC_Os07g22730 and LOC_Os03g18030 to the wounding were mitigated and enhanced in the mutants, respectively (Figure 7B). Moreover, among the 73 signaling-related DEGs, only LOC_Os06g11660 responded to the wounding more robustly in the SLs mutants (Figure 7C).

### 3.5. Conjoint Analysis of DEGs and DAMs 

**Figure 8:**
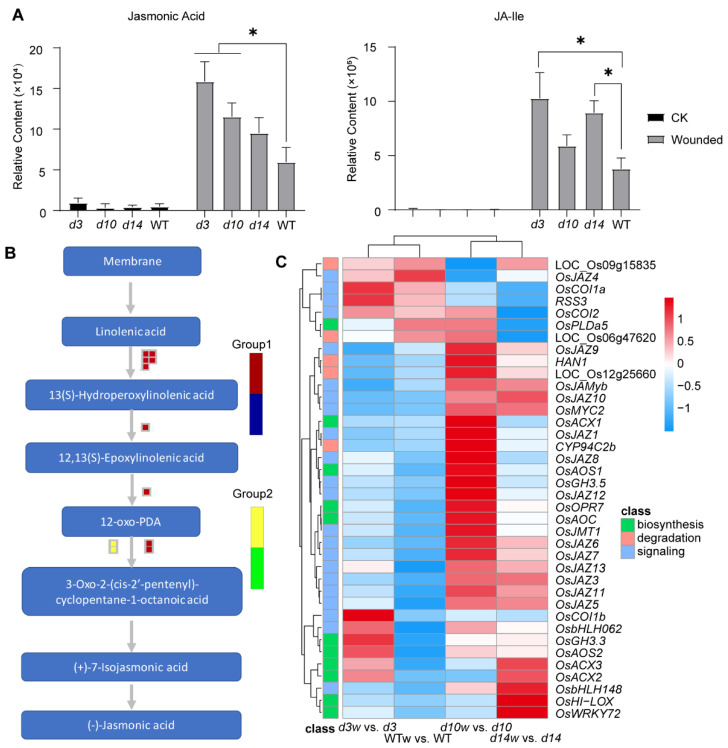
The crosstalk between JAs and SLs pathways. (**A**) The abundance of JA and JA-lle in *d10*, *d3*, *d14,* and wild-type plants. The data were represented as mean ± SD of two biological replicates. The Student’s *t*-test analysis indicated a significant difference (* *p* < 0.05). (**B**) MapMan-based “JA synthesis” of DEGs that regulated in three groups. The small heatmap in each section of the diagram shows the DEGs mapped to the pathway, and a small square indicates a transcript. Red and yellow indicate upregulated genes in each group; blue and green mean downregulated genes in each group. (**C**) Genes related to JAs synthesis, signaling, and degradation. Heatmaps of DEGs were drawn using the log_2_ (FC) value obtained from the pairwise comparison of samples. Red and blue indicate higher and lower fold change which scale by row.

According to metabolism analysis, despite relatively low contents under the normal condition, contents of JA and JA-lle in the SLs mutants were significantly higher than that in wild-type plants after wounding (Figure 2C and Figure 8A). To decode the molecular mechanism of how SLs affected the production of JAs and JA-lle, we mapped DEGs to a “JA synthesis” graph in MapMan (Figure 8B). The result showed that wounding triggered the expression of JAs biosynthetic genes (Figure 8B). Then, the documented genes in JAs biosynthesis, signaling, and degradation were further evaluated. A series of genes were upregulated by wounding with different fold changes in the SLs mutants and wild-type plants (Figure 8C, Table S6). Most of them responded to the wounding more robustly in the SLs mutants, especially biosynthesis-related genes (*OsAOS*1, *OsOPR*7, *OsAOC*, *OsJAR*2, *OsAOS*2, *OsACX*3, and *OsWRKY*72). In conclusion, SLs affected the expression of JAs pathway genes to regulate the accumulation of JAs and JA-lle upon wounding.

**Figure 9:**
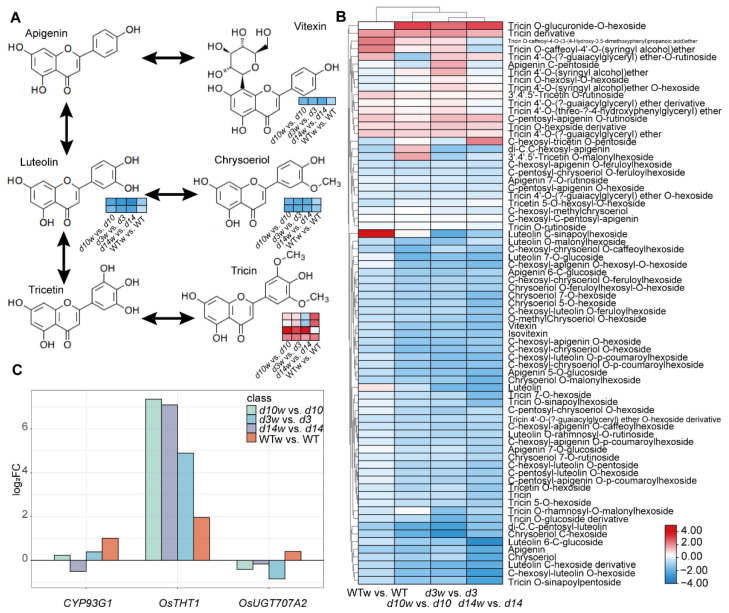
SLs and wound affected the flavones’ pathway. (**A**) DAMs were mapped to the flavone pathway. The small heatmap in each section of the diagram shows the DAMs mapped to the pathway, and a small square indicates a metabolite. (**B**) Heatmaps of flavones contents. (**C**) Expression levels of three genes related to flavones in SLs mutants and wild-type plants. Heatmaps were drawn using the log_2_ (FC) value obtained from the pairwise comparison of samples. Red and blue indicated upregulation and downregulation, respectively.

According to the metabolic analysis and pathways enrichment analysis, the SLs pathway inhibited the flavones’ responses to wounding. Then, we mapped DAMs to the flavones’ pathway (Figure 9A). Moreover, metabolome data showed that contents of most flavones declined after wounding, especially in SLs mutants more than wild-type plants (Figure 9B). We analyzed the expression of published and putative flavones’ synthetic genes and found three genes involved in SLs-regulated flavones metabolism (Figure 9C). *OsTHT1*, a hydroxycinnamoyl transferase gene inducing flavones’ accumulation and triggered by JAs and pathogen treatments [48], had a higher fold change in the SLs mutants upon wounding. However, *CYP93G1* (catalyzing the flavanones converted to flavones [49]) and *OsUGT707A2* (a flavone 5-O-glucosyltransferase gene [50]) were upregulated by wounding in the wild-type plants but displayed negative or no responses to wounding in the SLs mutants. The accumulation patterns of these transcripts resembled those of flavones. In conclusion, the SLs pathway affected the responses of flavones to wounding.

**Figure 10:**
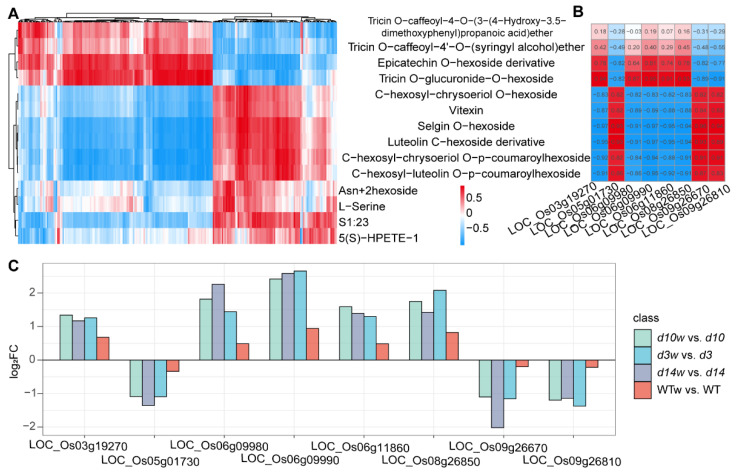
Candidate genes in SLs-inhibited flavones production. (**A**) Correlation coefficient clustering of DAMs and DEGs in Group 2 and Group 3. The horizontal axis represents DEGs. (**B**) Schematic representation of correlation coefficients between eight highly correlated genes and metabolites of flavones. Red represents positive correlation; blue represents negative correlation. Value in the color block was the correlation coefficient. (**C**) Log_2_ (FC) of eight genes in SLs mutants and wild-type plants.

Since the flavonoids were mainly represented in Group 2 and Group 3, we analyzed the correlation between DAMs and DEGs from Group 2 and Group 3 to identify genes in SLs-mediated wounding-regulated flavones (Table S7). There were three clusters in the correlation heatmap: (i) Cluster 1 was related to tricin O-caffeoyl-4-O-(3-(4-hydroxy-3.5-dimethoxyphenyl)propanoic acid), epicatechin O-hexoside derivative, tricin O-caffeoyl-4′-O-(syringyl alcohol)ether, and tricin O-glucuronide-O-hexoside; (ii) Cluster 2 contained luteolin C-hexoside derivative, selgin O-hexoside, C-hexosyl-luteolin O-p-coumaroylhexoside, C-hexosyl-chrysoeriol O-p-coumaroylhexoside, vitexin, and C-hexosyl-chrysoeriol O-hexoside; and (iii) Cluster 3 was constituted of other metabolites (Figure 10A). With a correlation threshold of 0.8, we identified eight genes in Cluster 2, which showed similar accumulation patterns with flavonoids (Figure 10B,C). *LOC_Os03g19270*, *LOC_Os06g09980*, *LOC_Os06g09990*, *LOC_Os06g11860*, and *LOC_Os08g26850* were positively regulated by wounding and expressed at higher levels in the SLs mutants. Meanwhile, *LOC_Os05g01730*, *LOC_Os09g26670*, and *LOC_Os09g26810* were negatively regulated by wounding only in the SLs mutants.

## 4. Discussion

Although the signaling transduction of wounding has been well documented, the metabolic re-programming under wounding remains to be explored on metabolome scales. In this study, we analyzed widely targeted metabolome and identified metabolomic responses to wounding. By using the SLs mutants, we characterized the role of SLs in wounding-induced metabolic re-programming.

Our work drew a whole picture of metabolic responses to wounding in rice. As well documented in many species, JAs responds to wounding drastically. In addition, wounding affected dozens of metabolites. For instance, tricin derivatives—a class of flavonoids—piled up upon wounded, coinciding with flavonoids’ defensive role [51,52]. Similarly, leaf herbivory induces a marked accumulation of flavonoids in tea [9]. However, the exact roles of wounding-responding flavonoids remain to be explored.

Our data evidenced crosstalk between SLs and JAs pathways. The analysis with the SLs mutants confirmed the participation of SLs in wounding responses. The impaired SLs pathway enhanced the accumulation of JA and JA-lle after wounding. A study on tomato defense against root-knot nematodes (RKNs) also provides evidence for the SLs-JAs crosstalk. Accumulation of JAs in response to RKN infection is enhanced by silencing of SLs biosynthetic genes and was suppressed by racGR24 [53]. Moreover, SLs signaling repressors (SMXL6 and SMXL7) directly interact with and accelerate the degradation of JAZs and amplify JAs signaling [33]. Our work implied that biosynthetic or catabolic genes of JAs could be essential for the crosstalk, whose responses to wounding were enhanced in the SLs mutants. The PPI network analysis proposed that *AOS*1 and its repressor, *WRKY*72, could be hub genes for wounding responses in the SLs mutants. That is, SLs and JAs interplay under wounding through complicated mechanisms, which need further efforts to elucidate.

JAs regulates the biosynthesis of many metabolites, including inducing the accumulation of cyanidin [54], luteolin, chrysoeriol, and apigenin [24,55]. The ethylene pathway has been reported to be activated by JAs to depress anthocyanins production and JA triggers the formation of flavone/isoflavone [24]. In this study, we also found wounding-induced ethylene accumulation (Figure 7A), which could promote the accumulation of luteolin [25]. In addition, SLs transcriptionally induce *Production of Anthocyanin Pigment1* (*PAP1*) and stimulate the production of anthocyanin in *Arabidopsis* [56]. In addition, compared with the wild-type rice, SLs mutants displayed mitigated wounding-affected expression of ethylene-related genes, implying an SLs–ethylene interaction on regulating the flavonoids pathway. We found that JAs repress some flavones production only when the SLs pathway was impaired. Our data suggested a complicated network through which JAs, ethylene, and SLs regulate flavonoids.

## 5. Conclusions

In conclusion, this study revealed the metabolomic response to wounding and the role of SLs. Our work discovered the SLs regulation on JAs accumulation and suggested a network of SLs-mediated metabolic responses to wounding. These results provide new insight into how plants metabolic adaptation and the SLs’ function.

## Data Availability

RNA sequence data that support the findings of this study have been deposited under SRA BioProject accession number PRJNA858785 and PRJNA622884.

## Supplementary Materials

The following are available online at: https://www.mdpi.com/article/10.3390/metabo12090789/s1, Table S1: The content of DAMs in each genotype; Table S2: RNA-seq data statistics; Table S3: TPM of differentially expressed genes in each genotype; Table S4: GO categories of DEGs; Table S5: KEGG pathway of DEGs; Table S6: TPM of JA biosynthesis, signaling, and degradation genes in each genotype; Table S7: Correlation coefficient of DAMs and DEGs in Group 2 and Group 3.
